# A Strip Cell in Pyroelectric Devices

**DOI:** 10.3390/s16030375

**Published:** 2016-03-15

**Authors:** An-Shen Siao, Ching-Kong Chao, Chun-Ching Hsiao

**Affiliations:** 1 Department of Mechanical Engineering, National Taiwan University of Science and Technology, No. 43, Keelung Rd., Sec. 4, Taipei 10607, Taiwan; qbasic147@gmail.com (A.-S.S.); ckchao@mail.ntust.edu.tw (C.-K.C.); 2 Department of Mechanical Design Engineering, National Formosa University, No. 64, Wunhua Rd., Huwei Township, Yunlin 632, Taiwan

**Keywords:** pyroelectricity, energy harvesting, dicing saw, temperature variation, lateral temperature gradient

## Abstract

The pyroelectric effect affords the opportunity to convert temporal temperature fluctuations into usable electrical energy in order to develop abundantly available waste heat. A strip pyroelectric cell, used to enhance temperature variation rates by lateral temperature gradients and to reduce cell capacitance to further promote the induced voltage, is described as a means of improving pyroelectric energy transformation. A precision dicing saw was successfully applied in fabricating the pyroelectric cell with a strip form. The strip pyroelectric cell with a high-narrow cross section is able to greatly absorb thermal energy via the side walls of the strips, thereby inducing lateral temperature gradients and increasing temperature variation rates in a thicker pyroelectric cell. Both simulation and experimentation show that the strip pyroelectric cell improves the electrical outputs of pyroelectric cells and enhances the efficiency of pyroelectric harvesters. The strip-type pyroelectric cell has a larger temperature variation when compared to the trenched electrode and the original type, by about 1.9 and 2.4 times, respectively. The measured electrical output of the strip type demonstrates a conspicuous increase in stored energy as compared to the trenched electrode and the original type, by of about 15.6 and 19.8 times, respectively.

## 1. Introduction

The ever-increasing demand for energy reflects an increase in human dependence on new 3C electronic products (“Cloud Computing”, “Connectivity Technology”, and “Client Device”). However, energy derived from fossil fuels is facing rapidly-depleted resources. In energy generation systems, output energy is the target to be maximized. In other words, efficient energy use must equate to maximum energy yield. Pyroelectric devices are widely used in energy harvesting applications [1,2,3,4]. Energy from a fluctuating heat source or temporal thermal variation has been successfully harvested using the pyroelectric effect. Harvesting of pyroelectric waste heat to power autonomous electronics has shown tremendous growth. Although thermoelectric generators have been the primary means of harvesting waste thermal energy from temperature gradients, they rely mainly on the Seebeck effect to convert a steady-state temperature difference at the junction of semiconductors or two different metals into an electromotive force or electrical energy. Moreover, thermoelectric devices cannot function at ambient temperatures with spatially-uniform and temporal temperature oscillations within short time periods [5,6].

Harvesting energy from the pyroelectric effect requires maintaining a temporal temperature variation. The pyroelectric effect is the characteristic of non-centrosymmetric dielectric materials which show a spontaneous electrical polarization as a function of temperature. Therefore, a temperature change in a set period of time decides a corresponding variation in the induced charge. When a temporal heat flux is applied to pyroelectric cells, the polar vector collapses and an electrical imbalance is generated on the surface of the cells. If the participating surfaces of the cells are insulated, an electrical potential is developed between them. Furthermore, if the surfaces of the cells are connected via a conducting medium, a flow of imbalanced charge, known as a pyroelectric current, occurs. Upon reducing the temperature, the dipoles are restored to their initial positions and the cycle can be repeated. In other words, when the temperature in the pyroelectric materials increases (dT/dt > 0), polarization decreases due to re-orientation of the dipole moment. It will generate an electrical current in an external circuit. On the contrary, when the pyroelectric materials are cooled (dT/dt < 0), polarization increases as dipoles gain their orientation. It will cause a current flow in reverse direction. This is the basis of pyroelectric energy harvesting and conversion. The induced current of the pyroelectric cells is based on the pyroelectric effect, which converts temporal temperature variations to corresponding electrical outputs. The pyroelectric current (I_p_) and charge (Q_p_) are given by [7]:

(1) $$I_{p}={\text{dQ}}_{p}/\text{dt}=\eta \times p\times A\times \text{dT}/\text{dt}$$

where η is the absorption coefficient of radiation; A is the electrode area; dT/dt is the temperature variation rate of the pyroelectric material and p is the pyroelectric coefficient of the pyroelectric material given by:

(2) $$p={\text{dP}}_{s}/\text{dT}$$

where P_s_ is the magnitude of the electrical polarization vector. A pyroelectric cell with a higher pyroelectric coefficient, applied where the temperature variation rate is higher, and where the electrode area is larger, will have an increased current under the same intensity of radiation per unit area. When the material and geometry of the cells are already established, the temporal temperature variation is the single deciding factor for enhancing the performance of pyroelectric harvesters. However, the temporal temperature variation in pyroelectric thin cells is hardly probed by experimental measurement. Hsiao *et al.* [8,9,10] used finite element models built by the commercial multi-physics software package COMSOL Multiphysics^®^ 5.0 (Stockholm, Sweden) to explore the temperature variation rate in PZT (Lead Zirconate Titantate) pyroelectric cells, with cavities produced by wet etching, trenches created by a precision dicing saw, and grooves produced by sandblast etching, all designed to improve the energy conversion efficiency of PZT pyroelectric harvesters by pyroelectricity. Moreover, Sharma *et al.* [5] used finite element analysis to compare seven different pyroelectric materials before suggesting the most efficient potential pyroelectric material. Sr_0.5_Ba_0.5_Nb_2_O_6_ (SBN) was found to be an excellent material in the performance about the maximum storage voltage of 11.47 V, the optimum power output of 4.9 μW and with the maximum stored energy of 576.87 μJ. Hsiao *et al.* [11] proposed a novel pyroelectric harvester integrating solar radiation with wind power to apply the pyroelectric effect to real situations. Solar radiation acts as the thermal source and wind acts as a dynamic source. A disk generator was used for harvesting the wind power. A planetary gear system was used to convert the rotary energy of the disk generator to drive a shutter for generating temporal temperature variations in pyroelectric cells. Siao *et al.* [12] discussed the geometry of pyroelectric cells coupled with various periods of temporal temperature variations to drive load resistance and store electrical energy. Although the thinner PZT cell presented an opportunity for enhancing the stored voltage, the performance of the much thinner 75 μm-thick PZT cell was frustrated due to the thinner cell with a larger electrical capacitance to decrease the induced voltage. The decrease in the induced voltage diminished the voltage difference between the PZT cell and the storage capacitor, in turn causing a reduction in the charge flow to the storage capacitor. In an effort to increase energy conversion efficiency, Nguyen *et al.* [3] used a dipping experiment to implement the Olsen cycle using a piston oscillating vertically and driving silicon oil back and forth between a heat source and a cold heat exchanger within a Teflon cylindrical chamber. Lee *et al.* [4] used a stamping experiment to perform the Olsen cycle by alternately placing a pyroelectric material for use in heat conduction with cold and hot sources. The various heat transfers were also shown to affect the efficiency of the Olsen cycle. Furthermore, Cuadras *et al.* [13] used various PVDF and PZT pyroelectric devices to convert time-dependent temperature variations into electrical current. They proposed pyroelectric materials as entropy sensors by estimating heat and temperature variations and further inferring the sensor entropy to the heat source entropy. The experimental results showed the pyroelectric devices could enable entropy monitoring of both electrochemical processes and irreversible thermodynamic processes to improve system performance.

Cyclic heat energy harvesting primarily uses the pyroelectric elements to convert temperature fluctuations into electricity. Although Hsiao *et al.* [8,9,10] used wet etching, sandblast etching, and a precision dicing saw to produce cavities and grooves to create lateral temperature gradients [14] for enhancing temperature variation rates, the trenched or etched electrode increased the PZT cell capacitance to reduce the induced voltage, which resulted from trimming the thickness of the cell. Therefore, reducing the thickness of pyroelectric cells to enhance energy conversion efficiency due to a larger cell capacitance is not a suitable approach. It is desirable to retain cell thickness to increase the temperature variation rates. In the present study, a PZT cell with a strip form was used to increase temperature variation rates induced by lateral temperature gradients without an increase in the PZT cell capacitance for improving the performance of PZT pyroelectric harvesters; the strip PZT cell with a high-narrow cross section was then fabricated via a precision dicing saw.

## 2. Materials and Methods

### 2.1. Design and Simulation for Strip PZT Pyroelectric Cells

**Figure 1:**
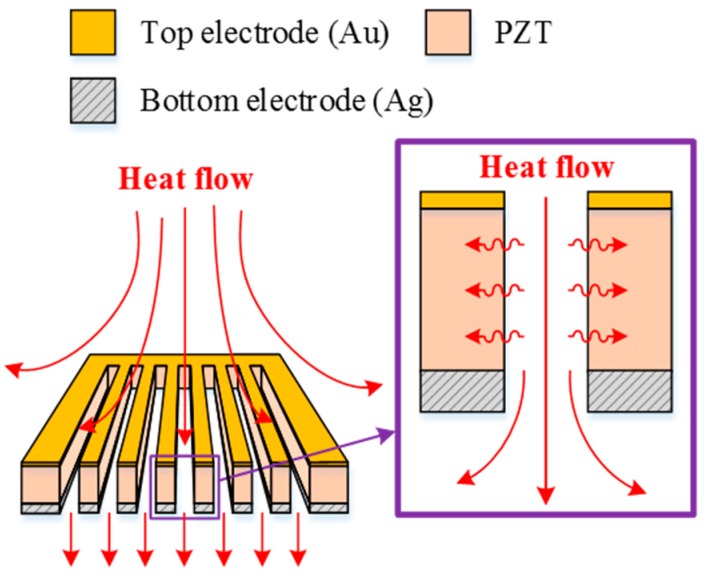
Schematic diagram for describing operations of the strip PZT pyroelectric cell.

**Figure 2:**
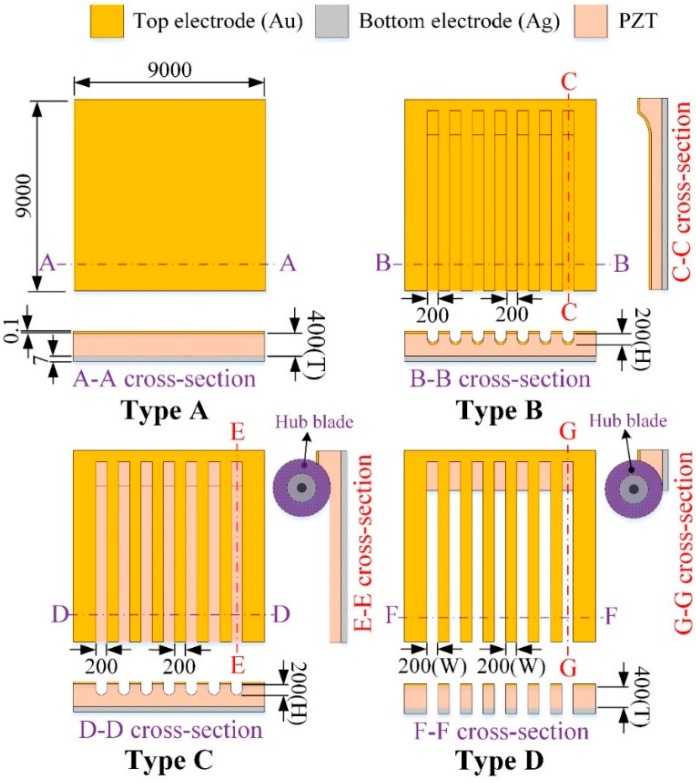
The pyroelectric PZT cells with various structures (unit: μm).

A PZT pyroelectric cell with a dimension of 9 mm × 9 mm × 0.414 mm was used. The cell was comprised of a 0.4 mm-thick PZT sheet sandwiched between a top and bottom electrode. The electrodes consisted of a silver film 7 μm thick. PZT samples were provided by Eleceram Technology Co., Lu Jwu Hsiang, Taiwan. The geometry and properties of the commercial PZT pyroelectric sheet are tabulated in Table 1. Although a thick PZT cell with a low electrical capacitance is more likely to generate higher voltages than a thin PZT cell with a high electrical capacitance, a thick PZT cell possesses a low temperature variation rate due to a large thermal capacity under heat radiation uniformly applied to the top surface of the PZT cell; this is flawless in pyroelectric cells with a low electrical capacitance and a high temperature variation rate for application as pyroelectric harvesters. When pyroelectric materials are decided, increasing the thickness of pyroelectric cells is advantageous in reducing cell capacitance. Although decreasing the electrode area can reduce cell capacitance, the induced current of the cells will be diminished. Increasing the temperature variation rate in the thicker PZT cell is troublesome due to the larger thermal capacity. A strip cell was proposed to alleviate these difficulties and enhance the performance of the PZT pyroelectric harvesters. The cross section of the strip cell was a high-narrow form, as depicted in Figure 1. This form can induce the lateral temperature gradient via the side walls of the strips to further increase the temperature variation rate in the thicker PZT cell. The strip was fabricated using a precision dicing saw (DS-150 II, Everprecision Tech Co., Ltd., New Taipei city, Taiwan). The width of the strips (W), about 200 μm, was controlled by a hub blade, and the depth of the strips (T), about 400 μm, was the thickness of the PZT cell. Moreover, the depth of the trenches was controlled by the use of a dicing saw machine. The cutting depth (H) in the trenches was fixed at 200 μm. Four structures in the PZT cell were compared with each other to improve the energy conversion efficiency of the PZT pyroelectric harvester, as depicted in Figure 2. Type A is the original PZT cell, Type B is the PZT cell with the trenched electrode, Type C is the PZT cell with the trenches. and Type D is the strip PZT cell.

**Figure 3:**
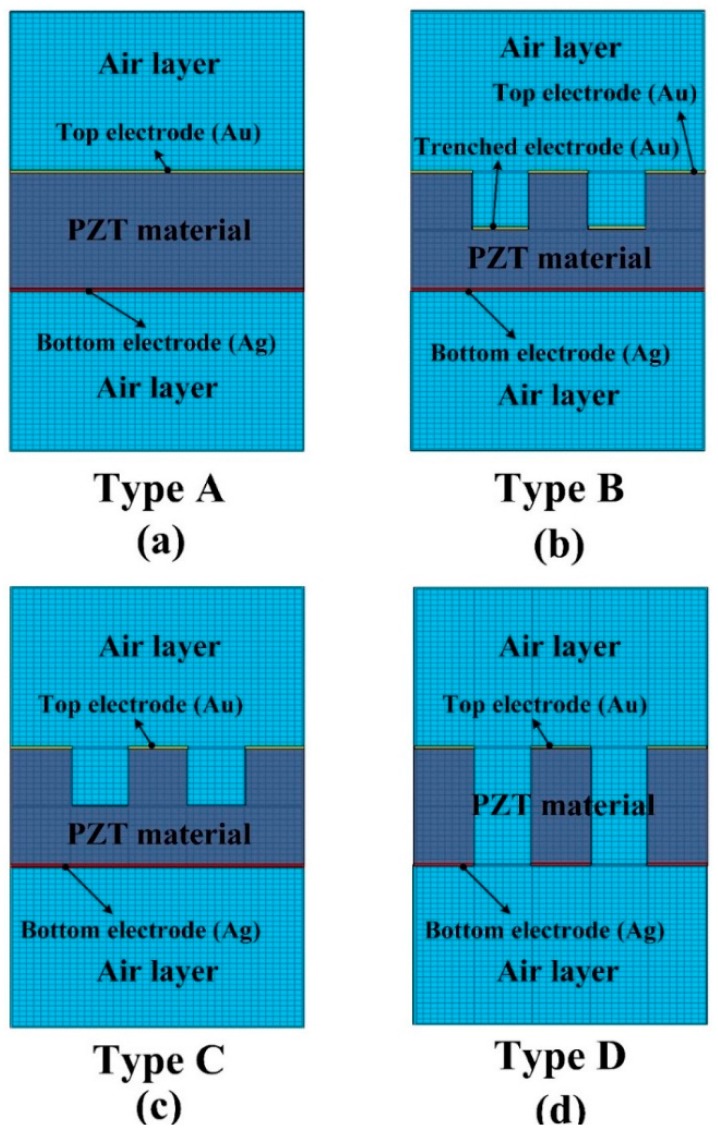
Finite element model for the PZT pyroelectric cells with various structures. (**a**) Type A; (**b**) Type B; (**c**) Type C; (**d**) Type D.

A two-dimensional finite element model was constructed by the commercial multi-physics software COMSOL Multiphysics^®^ 5.0 (Stockholm, Sweden) to probe the temperature variation rate in the PZT pyroelectric cells with various structures (Types A to D). The model solved the transient temperature fields for the PZT pyroelectric cells and the air flow in a rectangular zone by using the heat transfer module. Thermal energy was transported via conduction in the PZT pyroelectric cell, and through conduction and convection in a heating and cooling air flow. The temperature field was continuous across the internal surfaces between the PZT pyroelectric cell and the air. The incident heat irradiation with a square waveform, various frequencies, an air flow rate of about 0.5 m/s and temperature fluctuations between 50 °C to 150 °C was applied to the top side of the air layer. The thermal properties and parameters of the materials used in the model are listed in Table 2. There was an isotropic and time-independent assumption for the PZT pyroelectric cell and the electrodes in the model. The thermal conductivity, heat capacity, and air density were all temperature-dependent material properties, and set according to the COMSOL software. The models with four structures were meshed by a regular mesh, as shown in Figure 3.

### 2.2. Fabrication and Measurement for Strip PZT Pyroelectric Cells

**Figure 4:**
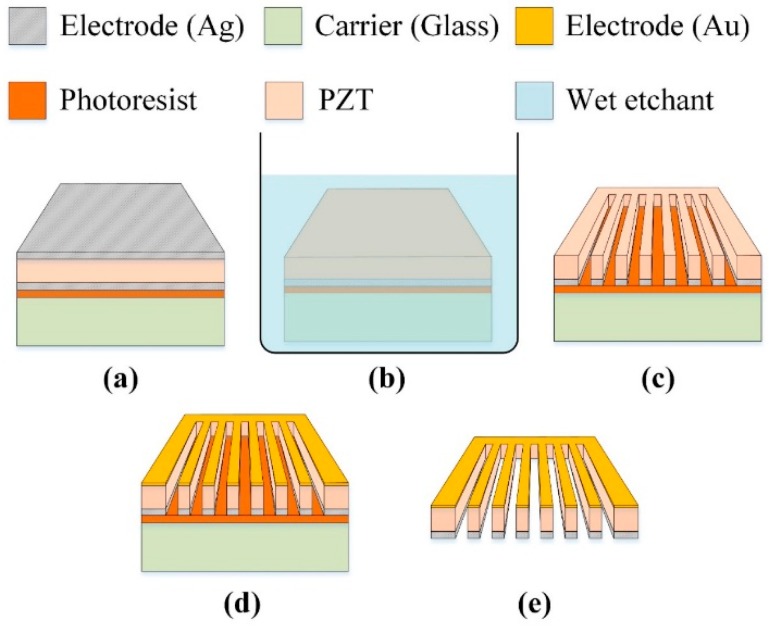
Process flow of the strip PZT pyroelectric cell. (**a**) The PZT cell attached to a glass carrier; (**b**) the top electrode (Ag) etched; (**c**) the PZT cell stripped; (**d**) the top electrode (Au) deposited on the strip PZT pyroelectric cell; (**e**) the carrier removed.

**Figure 5:**
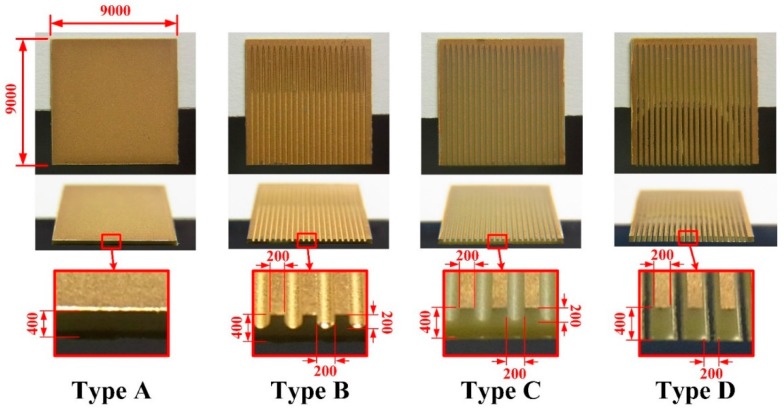
Fabricated PZT pyroelectric cells with various structures (unit: μm).

The strip cell was adopted to promote the temperature variations in the thicker PZT pyroelectric cell with the lower capacitance. The fabrication flow for the strip PZT cell is depicted in Figure 4. The PZT pyroelectric cell was firstly attached to a glass carrier, as shown in Figure 4a. A wet etchant with a recipe of HNO_3_-H_2_O = 7:3 was used to remove the fully covered top electrode of the PZT cells, as shown in Figure 4b. A precision dicing saw machine was used to fabricate the trenches and the strips in the PZT cells, as shown in Figure 4c. Furthermore, a 100 nm-thick gold film was deposited on the top side of the PZT cells using an E-beam evaporator to produce the top and trenched electrodes, as shown in Figure 4d. Finally, the glass carrier was removed to terminate the fabrication of the PZT pyroelectric cells, as shown in Figure 4e. The fabricated PZT pyroelectric cells with various structures are depicted in Figure 5.

**Figure 6:**
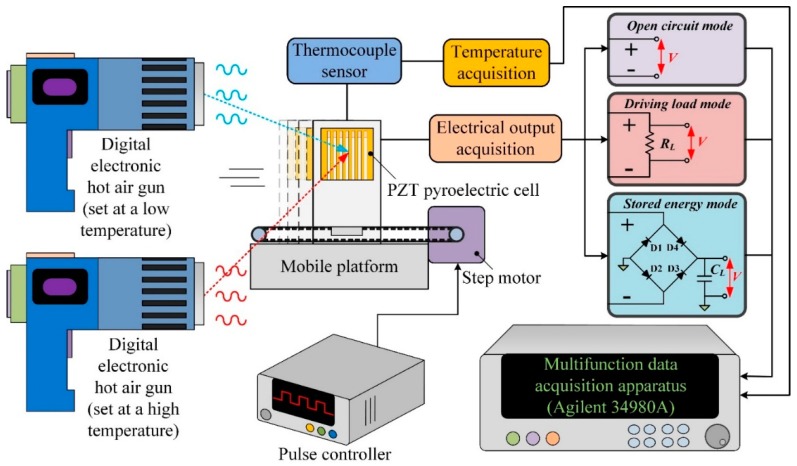
Measurement setup for the PZT pyroelectric harvester.

An integral system coupling thermal and electrical measurements was set to evaluate the performance of the present PZT cells with various structures, as depicted in Figure 6. The measurement setup consisted of two hot air guns, a homemade mobile platform, a step motor, a jig, a thermocouple sensor, and the PZT cells. Two hot air guns with a digital electronic control were used as thermal sources to produce temperature fluctuations ranging from 50 °C to 150 °C. Moreover, the mobile platform driven by the step motor controlled with a pulse controller was used to produce temporal temperature variations (dT/dt) during different time periods. The jig set on the mobile platform was used to hold the PZT cells along the borders in order to completely expose it to the air. The distance between the guns and the PZT pyroelectric cell was about 40 mm. The temperature in the PZT cell was probed using type K (Chromel/Alumel) thermocouple sensors, which were attached to the bottom electrode near the center of the PZT pyroelectric cell to ensure fine thermal contact. Eventually, the outputs of temperature, current, and voltage were simultaneously measured with a computer-controlled data acquisition apparatus (Agilent 34980A, Santa Clara, CA, USA). Furthermore, the PZT pyroelectric cells with various structures were tested to determine the power and charge of each cell. The charge generated from temporal temperature variations was stored in a capacitor C_L_ as a storage device using the full-wave rectifier circuit. The measured forward voltage drop of the diodes (Model: 1N4148) was 0.62 V. C_L_ was a 4.7 μF electrolytic capacitor with a 50 V maximum voltage.

## 3. Results and Discussion

**Figure 7:**
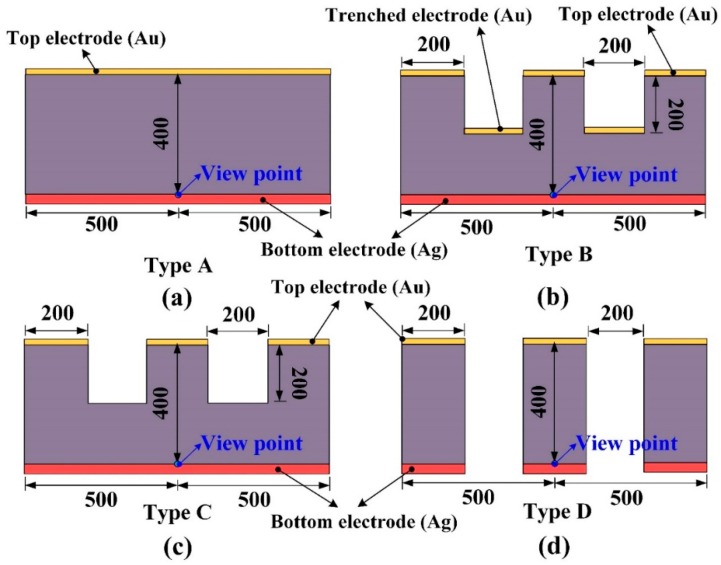
Points defined in the PZT cells with various structures (Types A to D) (unit: μm). (**a**) Type A; (**b**) Type B; (**c**) Type C; (**d**) Type D.

**Figure 8:**
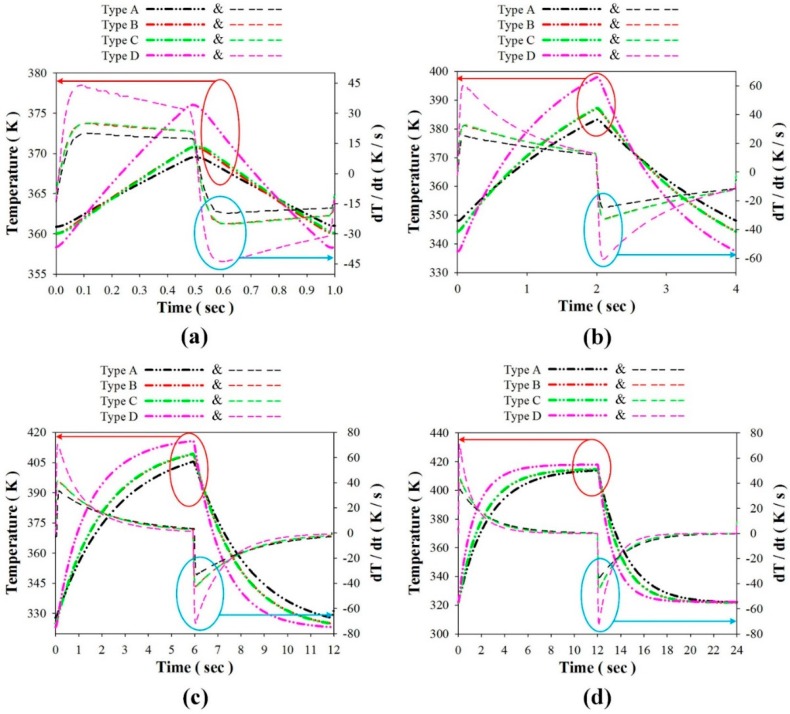
Temperature variation rate as a function of the temperature and time for using the PZT cells with various structures (Types A to D) and periods of about (**a**) 1 s; (**b**) 4 s; (**c**) 12 s; and (**d**) 24 s.

**Figure 9:**
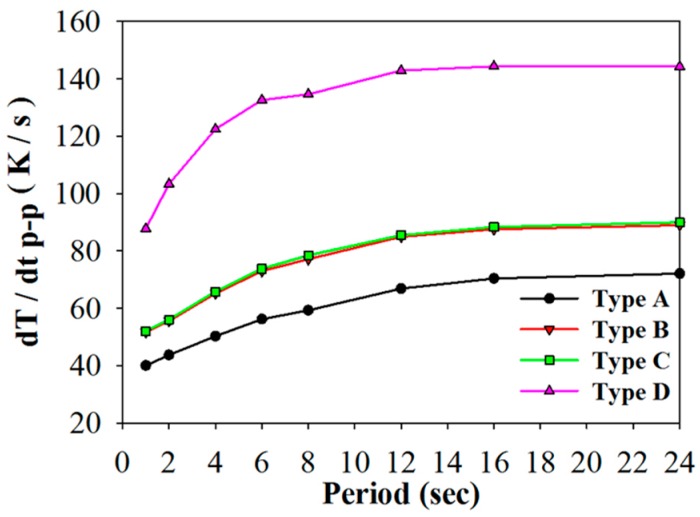
Relationship between the peak to peak temperature variation rate and period for using the PZT cells with various structures.

Controlled temperature oscillations were applied to the PZT pyroelectric cells of various structures to assess temperature variation rates and electrical outputs via the simulation and the experiment. In the simulation results, the temperature variation rate increased when the point approached the top side of the PZT layer because the incident radiation power approached the top electrode. Adopting the temperature variation rate at the point near the top electrode to distinguish the properties of the PZT cells with various structures (Types A to D) was a failure. A worst-case scenario, using the points possessing the lowest temperature variation rates, was used to differentiate between the PZT cells with various structures. These points in the PZT layers were put in the middle of the top electrode and near the bottom electrode, as depicted in Figure 7. Moreover, Hsiao *et al.* [8,9,10,12] had used the finite element method for discussing the temperature variation rates in the PZT cells with the trenched structure. Therefore, the temperature variation rates of the PZT layers at the point near the bottom electrode and in the middle of the top electrode were adopted to estimate the performance of the PZT cells with various structures. Figure 8 shows the temperature variation rate as a function of the temperature and time in using the PZT cells of varying structures and periods. It was obvious that the period increased when the temperature variation rate and temperature of the PZT cells increased due to the 400 μm thick PZT layer with the larger thermal capacity. Figure 9 shows the relationship between the peak-to-peak temperature variation rate and the period for using the PZT cells with various structures. The maximum peak-to-peak temperature variation rate was 144.3 K/s in Type D with the period of 24 s. The peak-to-peak temperature variation rate of Type D was 2.4, 1.9, and 2.0 times that of Types A–C, respectively.

The pyroelectric device is usually considered a source of current in parallel with its equivalent capacitance (*C_p_*) and resistance (*R_p_*) [1,15,16]. In the experiment results, an LCR meter (Agilent E4980A, Santa Clara, CA, USA) was first used to measure the capacitance and resistance of the PZT cells with various structures at l KHz and at room temperature. Table 3 shows the electrode area, capacitance and resistance of the PZT cells with various structures. It was obvious that the electrode area was the largest at Types A and B. The capacitance was the lowest at Type D due to the smallest electrode area without the trenched electrode and the trenches, but it was the largest at Type B due to the trenched electrode and the trenches trimming the thickness of the PZT cell. The capacitance of Type D was lower than that of Types A–C; about 38%, 58%, and 10%, respectively. Moreover, the resistance of Type B was the smallest due to the trenched electrode and the trenches trimming the thickness of the PZT cell. The resistance of Type D was the largest due to the strips removing the PZT material; Type D also had the smallest thermal capacity for favorably increasing temperature variation rates in the PZT pyroelectric cells. Therefore, the strip PZT cell had lower capacitance for advantageously enhancing the induced voltage, the larger resistance for reducing the internal electrical consumption of the cell, and the lower thermal capacity for increasing the temperature variation rates.

**Figure 10:**
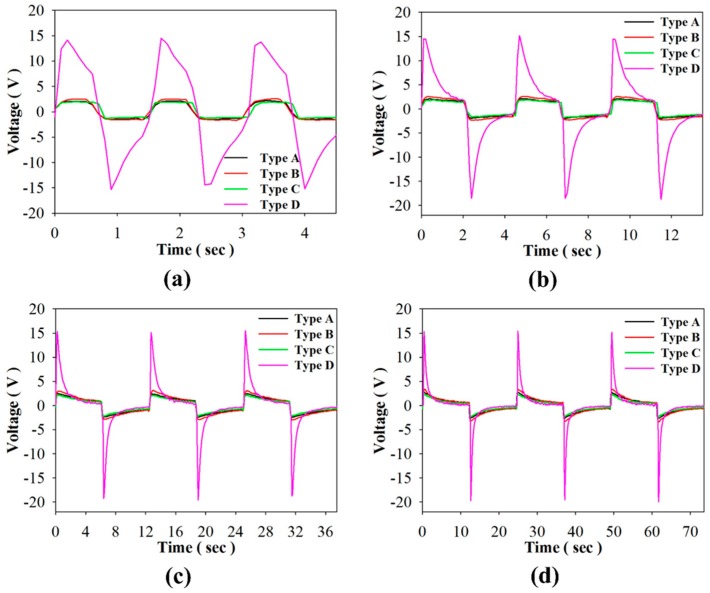
Relationship between the induced voltage and time for using the PZT cell with various structures (Types A to D) and periods of about (**a**) 1 s; (**b**) 4 s; (**c**) 12 s; and (**d**) 24 s.

**Figure 11:**
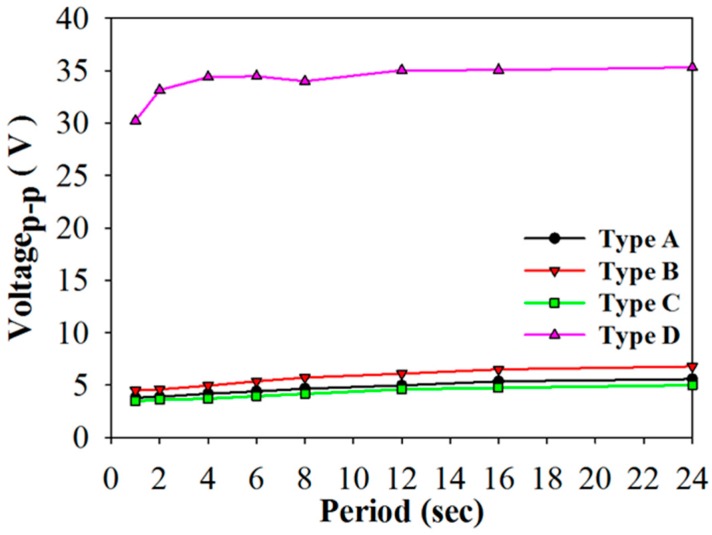
Relationship between the induced peak to peak voltage and period for using the PZT cell with various structures.

In the open circuit mode, Figure 10a–d show the relationship between the induced voltage and time when using PZT cells of varying structures and periods. Figure 11 shows the relationship between the induced peak to peak voltage and period for using the PZT cells with various structures. The induced voltage increased when the period increased. The maximum induced peak to peak voltage was 35.3 V in Type D with a period of 24 s. The induced peak to peak voltage of Type D was 6.3, 5.2, and 7.1 times that of Types A–C, respectively. Moreover, the induced peak to peak voltage in Type B was 1.2 times that found in the original PZT cell. This result had been proven in reference [5]. The induced peak to peak voltage in Type C was the lowest due to the smaller electrode area. Furthermore, the induced voltage was smaller in the lower periods due to the 400 μm thick PZT cell with a larger thermal capacity. In other words, the thicker PZT cells needed more time to absorb the thermal energy. Furthermore, the induced peak to peak voltage of Types A–C was about 5 V. Voltage_p-p_ of Types A–C would lose near 50% in the forward voltage drop of 2.48 V while the full-wave rectifier was used in the stored energy mode, but Voltage_p-p_ of Type D would only lose about 7%.

**Figure 12:**
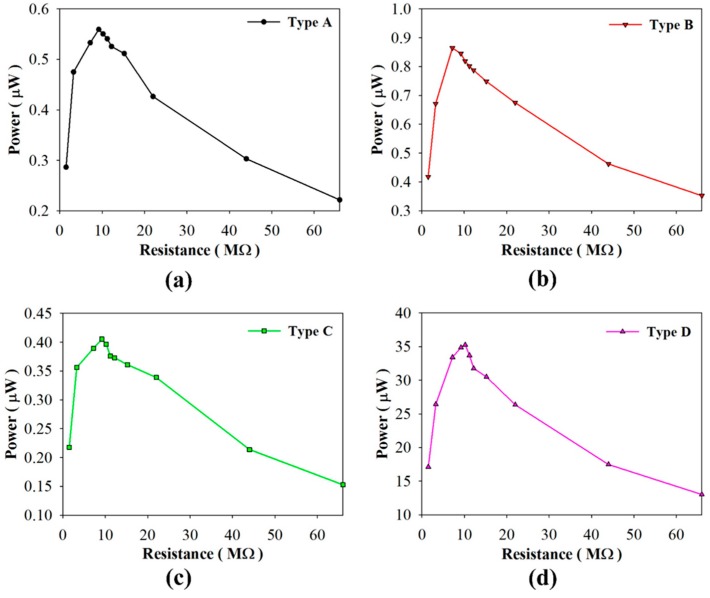
Harvested power as functions of the load resistance when using the PZT cells of various structures and the period of 4 s. (**a**) Type A; (**b**) Type B; (**c**) Type C; (**d**) Type D.

Considering that the PZT pyroelectric cells directly drive load resistances, Figure 12 shows the harvested power as a function of the load resistance when using PZT cells of varying structures and a period of 4 s. All types had the optimal harvested power at a load resistance of about 9 MΩ. The maximum harvested power was 35.26 μW in Type D with the period of 4 s. It was evident that the harvested power of Type D was 63, 41, and 87 times that of Types A–C, respectively. Moreover, the harvested power of Type B was 1.5 times that of Type A. Hence, the strip PZT cell possessed the maximum harvested power.

**Figure 13:**
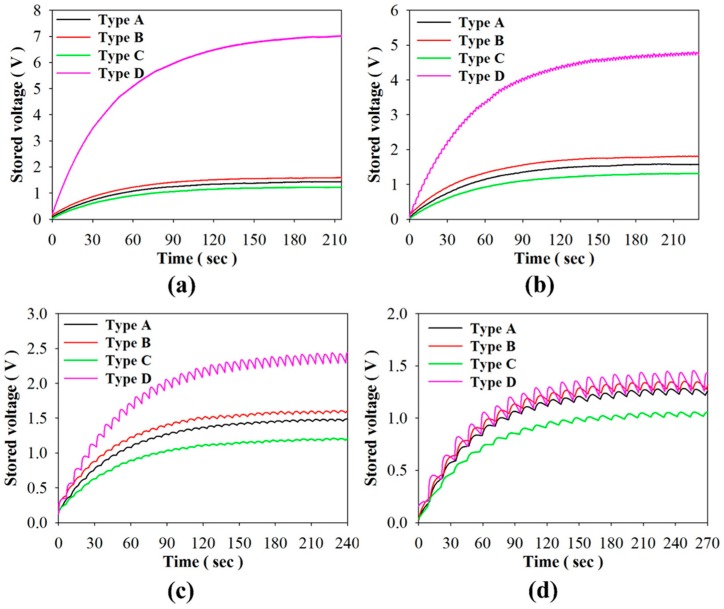
Relationship between the stored voltage and time when using the PZT cells with various structures (Types A to D) and periods of about (**a**) 1 s; (**b**) 4 s; (**c**) 12 s; and (**d**) 24 s.

**Figure 14:**
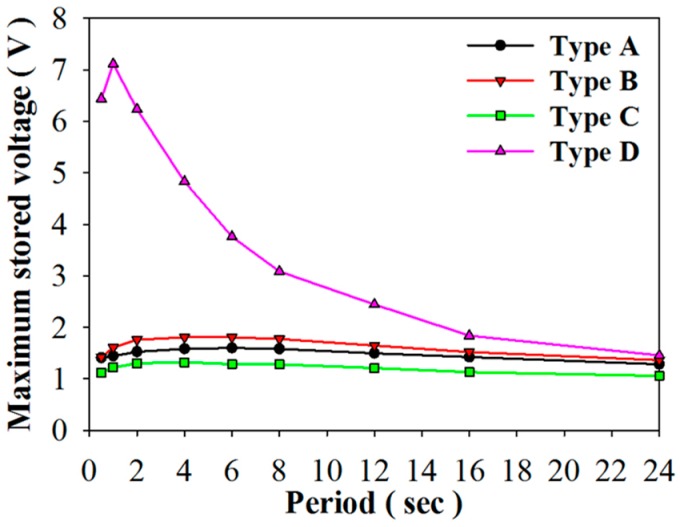
Relationship between the maximum stored voltage and period for using the PZT cells with various structures.

As for the stored energy mode, Figure 13a–d show the relationship between the stored voltage and time when using PZT cells of varying structures and periods. Figure 14 shows the relationship between the maximum stored voltage and period for using PZT cells with various structures. Stored voltage is defined as a voltage when the induced charge generated from a pyroelectric cell with temporal temperature variations is adopted to store the electrical energy in a 4.7 μF electrolytic capacitor. The optimal periods to maximize the stored voltage were situated at about 6 s, 4 s, 4 s, and 1 s when using PZT cells with various structures, Types A–D, respectively. The optimal period was the smallest at 1 s in Type D. The stored energy of Type D was greatly dependent on the period. The maximum stored voltage and energy were, respectively, 7.1 V and 118.5 μJ in Type D with a period of 1 s. The maximum stored voltage of Type D was 4.4, 3.9, and 5.5 times that of Types A–C, respectively. The maximum stored energy of Type D was 19.8, 15.6, and 29.6 times that of Types A–C, respectively. Hence, Type D was used to maximize the temperature variation rate for further enhancing the stored energy and voltage by the lateral temperature gradients of the strips. Furthermore, the performance of Type D was acutely dependent on the period due to the lower thermal capacity and the larger temperature variation rate. In addition, the ripples in Figure 13 were related to temperature variations with various time periods. In the larger period, the temperature variation rates could not hold at the maximum value in the whole period. This resulted the discharge of the stored voltage. While the period decreased, the peak temperature variation rates were continuously generated. Hence, the ripples in the smaller period were obscure and the stored voltage greatly increased.

## 4. Conclusions

This study utilized the pyroelectric effect for thermal energy harvesting using PZT cells with various structures. The strip PZT pyroelectric cell, fabricated by a dicing saw machine, displays some advantages by using lateral temperature gradients to increase the temperature variation rates, and retaining the cell thickness to hold the smaller cell capacitance in order to further increase the induced voltage. Both the experiment and the simulation showed that the strip cell improves the electrical output and enhances energy conversion efficiency. The pyroelectric cell with the strip type has a larger temperature variation rate than both the trenched electrode and the original type, by about 1.9 and 2.4 times, respectively. The measured electrical output of the strip type exhibits an obvious increase in the amount of stored energy as compared to the trenched electrode and the original type; about 15.6 and 19.8 times, respectively.

## Figures and Tables

**Table 1:** Geometry and properties of the commercial PZT pyroelectric sheet.

Item	Data
**Size (mm × mm)**	9 × 9
**Thickness (μm)**	400
**Area (mm^2^)**	81
**Relative dielectric constant (ε_33_^T^/ε_0_)**	2100
**Density (g/cm^3^)**	7.9
**Poling field (V/μm)**	3.5
**Pyroelectric coefficient (10^−4^ C·m^−2^·K^−1^)**	6.97

**Table 2:** Material parameters for finite element analysis.

Material	Density (g/cm^3^)	Thermal Conductivity (W/(m·K))	Specific Heat (J/(g·K))
PZT sheet	7.9	2.1	0.36
Electrode(Au)	19.3	317	0.129
Electrode(Ag)	10.49	429	0.24
Air	Eq1(T_abs_)	Eq2(T_abs_)	Eq3(T_abs_)
Eq1(T_abs_) = 101.325×10^3^ × 0.02897/8.314/T_abs_			
Eq2(T_abs_) = 0.00227583562 + 1.15480022 × 10^−4^ × T_abs_ − 7.90252856×10^−8^ × T_abs_^2^ + 4.11702505 × 10^−11^ × T_abs_^3^ − 7.43864331 × 10^−15^ × T_abs_^4^			
Eq3(T_abs_) = 1.04763657 − 3.72589265 × 10^−4^ × T_abs_ + 9.45304214 × 10^−7^ × T_abs_^2^ − 6.02409443 × 10^−10^ × T_abs_^3^ + 1.2858961 × 10^−13^ × T_abs_^4^			

Note: T_abs_: Absolute temperature.

**Table 3:** Impedance measurement for the PZT cells with various structures.

Item	Type A	Type B	Type C	Type D
Electrode area (mm^2^)	81	81	44.5	44.5
Capacitance, C_p_ (nF)	4.3	6.4	3.0	2.7
Resistance, R_p_ (MΩ)	2.0	1.3	2.6	3.1
